# Assessment of ChatGPT-3.5's Knowledge in Oncology: Comparative Study with ASCO-SEP Benchmarks

**DOI:** 10.2196/50442

**Published:** 2024-01-12

**Authors:** Roupen Odabashian, Donald Bastin, Georden Jones, Maria Manzoor, Sina Tangestaniapour, Malke Assad, Sunita Lakhani, Maritsa Odabashian, Sharon McGee

**Affiliations:** 1 Department of Oncology, Barbara Ann Karmanos Cancer Institute, Wayne State University Detroit, MI United States; 2 Department of Medicine, Division of Internal Medicine, The Ottawa Hospital and the University of Ottawa Ottawa, ON Canada; 3 Mary A Rackham Institute, University of Michigan Ann Arbor, MI United States; 4 Department of Plastic Surgery, University of Pittsburgh Medical Center Pittsburgh, PA United States; 5 Department of Medicine, Division of Internal Medicine, Jefferson Abington Hospital Philadelphia, PA United States; 6 The Ottawa Hospital Research Institute Ottawa, ON Canada; 7 Department of Medicine, Division of Medical Oncology, The Ottawa Hospital and the University of Ottawa Ottawa, ON Canada; 8 Cancer Therapeutics Program, Ottawa Hospital Research Institute Ottawa, ON Canada

**Keywords:** artificial intelligence, ChatGPT-3.5, language model, medical oncology

## Abstract

**Background:**

ChatGPT (Open AI) is a state-of-the-art large language model that uses artificial intelligence (AI) to address questions across diverse topics. The American Society of Clinical Oncology Self-Evaluation Program (ASCO-SEP) created a comprehensive educational program to help physicians keep up to date with the many rapid advances in the field. The question bank consists of multiple choice questions addressing the many facets of cancer care, including diagnosis, treatment, and supportive care. As ChatGPT applications rapidly expand, it becomes vital to ascertain if the knowledge of ChatGPT-3.5 matches the established standards that oncologists are recommended to follow.

**Objective:**

This study aims to evaluate whether ChatGPT-3.5’s knowledge aligns with the established benchmarks that oncologists are expected to adhere to. This will furnish us with a deeper understanding of the potential applications of this tool as a support for clinical decision-making.

**Methods:**

We conducted a systematic assessment of the performance of ChatGPT-3.5 on the ASCO-SEP, the leading educational and assessment tool for medical oncologists in training and practice. Over 1000 multiple choice questions covering the spectrum of cancer care were extracted. Questions were categorized by cancer type or discipline, with subcategorization as treatment, diagnosis, or other. Answers were scored as correct if ChatGPT-3.5 selected the answer as defined by ASCO-SEP.

**Results:**

Overall, ChatGPT-3.5 achieved a score of 56.1% (583/1040) for the correct answers provided. The program demonstrated varying levels of accuracy across cancer types or disciplines. The highest accuracy was observed in questions related to developmental therapeutics (8/10; 80% correct), while the lowest accuracy was observed in questions related to gastrointestinal cancer (102/209; 48.8% correct). There was no significant difference in the program’s performance across the predefined subcategories of diagnosis, treatment, and other (*P*=.16, which is greater than .05).

**Conclusions:**

This study evaluated ChatGPT-3.5’s oncology knowledge using the ASCO-SEP, aiming to address uncertainties regarding AI tools like ChatGPT in clinical decision-making. Our findings suggest that while ChatGPT-3.5 offers a hopeful outlook for AI in oncology, its present performance in ASCO-SEP tests necessitates further refinement to reach the requisite competency levels. Future assessments could explore ChatGPT’s clinical decision support capabilities with real-world clinical scenarios, its ease of integration into medical workflows, and its potential to foster interdisciplinary collaboration and patient engagement in health care settings.

## Introduction

OpenAI released ChatGPT, a pioneering artificial intelligence (AI) language model, in late 2022. ChatGPT-3 is an AI chatbot that can comprehend user input and react to it in a manner that is natural and human-like [1]. The program was trained on a large body of data sourced from the internet, including textbooks, articles, social media posts, and web-based forums, up to the last quarter of 2021 [2]. It works by analyzing user input text to generate a response using a probabilistic distribution of words and phrases derived from its training data. To date, it has significantly impacted numerous disciplines, including law, health care, and medical education [3-6]. Large language models like ChatGPT-3.5 represent a significant advancement in the preceding class of deep learning–based models, by facilitating the interpretation, processing, and production of natural language [7].

The use of AI has rapidly emerged as a promising approach in the health care industry, where it has been applied to medical imaging analysis, drug discovery, and patient monitoring [8]. Recent research has evaluated ChatGPT-3.5’s abilities to respond to standardized questions from professional examinations for law and the United States Medical Licensing Examination (USMLE) [3,4]. ChatGPT-3.5 was able to achieve passing grades on these examinations while providing logical and informative explanations. Additionally, studies have been conducted to assess ChatGPT’s capabilities in responding to international medical licensing examinations from countries such as Italy, France, Spain, the United Kingdom, and India. The success rates observed ranged between 22% and 73% [9].

AI and Chat GPT showcase substantial promise in augmenting medical consultations, offering preliminary diagnostic suggestions, and providing a vast knowledge base for medical practitioners and patients alike [10]. However, while it embarks on a path toward a more integrated health care AI system, several limitations hinder its full potential. The model’s reliance on historical data without the ability to access real-time patient data can lead to outdated or inaccurate information dissemination. Additionally, its inability to comprehend nuanced human emotions and the ethical implications surrounding patient data privacy remain significant hurdles [11].

AI has displayed a notable deficiency in grasping context and nuance, elements that are fundamental for delivering safe and effective patient care [12]. Furthermore, analyzing the prospects of job automation in health care, Frey and Osborne [13] have projected that while administrative roles within the sector, such as health information technicians, exhibit a high likelihood of automation at 91%, the odds plummet to a mere 0.42% for the automation of roles held by physicians and surgeons. This stark contrast underscores the intricate nature of medical practice, which extends beyond the mere application of codified knowledge. Additionally, there is a burgeoning discussion around the ethical dimensions of using conversational AI in medical practice. The crux of the issue revolves around the substantial volume of high-quality data required to train these models. Present-day algorithms are often honed on biased data sets, inheriting not just the availability, selection, and confirmation biases inherent in the data but also displaying a propensity to exacerbate these biases [14]. Looking ahead, the evolving capabilities of AI hint at the potential for tackling more sophisticated tasks, such as orchestrating experiments or future clinical trials [15] or engaging in peer review processes [16].

The American Society of Clinical Oncology Self-Evaluation (ASCO-SEP) program created a comprehensive educational program to help physicians keep up to date with the many rapid advances in the field. The question bank consists of multiple choice questions (MCQs) addressing the many facets of cancer care, including diagnosis, treatment, and supportive care. It is intended to evaluate participants’ knowledge and give them feedback to direct future learning. The program is largely regarded as the leading resource for cancer specialists seeking to gain and maintain professional licensure in the field of medical oncology [17].

However, the evolving complexities of oncological care necessitate additional tools that can aid oncologists in clinical decision-making. By assessing ChatGPT-3’s ability to answer ASCO-SEP questions, this study’s objective is to understand ChatGPT’s potential to serve as a supportive instrument in clinical decisions, offering instantaneous insights for health care providers, and to identify novel and efficient educational aids in oncology, with a specific emphasis on their role in clinical decisions.

## Methods

### Input Data

**Figure 1:**
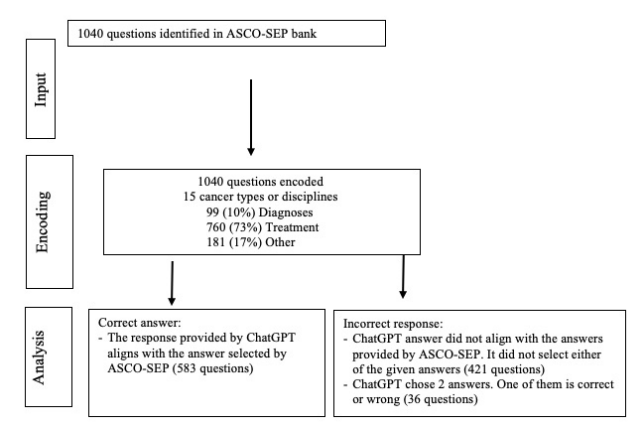
Schematic of sourcing, encoding, and scoring procedures. ASCO-SEP: American Society of Clinical Oncology Self-Evaluation Program.

Questions were sourced from ASCO-SEP, which consists of approximately 1000 MCQs covering the spectrum of cancer care. The question bank was accessed from February 2023 to March 2023. As ChatGPT-3.5 can only generate responses to textual data, the study excluded questions with images, tables, or other non-textual content. Questions consisted of an information stem followed by a specific question with 3-5 possible answers (A-E), along with their corresponding letter choices, only 1 of which was correct. Figure 1 illustrates the workflow for data sourcing, input, encoding, and analysis.

Before proceeding with the analysis, a random spot check was performed. For this, a random subset of the ASCO-SEP questions was selected, and their answers, explanations, or related content were manually cross-referenced with Google’s index to ensure that they were not present before January 1, 2022, the last date accessible to the ChatGPT training data.

During this study, we used the free version of ChatGPT-3.5. At that time, ChatGPT-4 and its associated plugins were not yet available.

### Encoding

We imported individual ASCO-SEP questions, including the information stem and multiple-choice response options, into the ChatGPT-3.5 interface. The questions were formatted to include the question stem, followed by each potential response on a separate line. We did not change the structure of the questions given to ChatGPT-3.5 and entered them in the original format provided by ASCO-SEP without altering the phrasing or the wording. A new conversation session was started in ChatGPT for each question. We did not provide ChatGPT-3.5 with any prompts and offered only one opportunity to answer each question.

### Data Analysis

Selected questions were grouped by cancer type or discipline (eg, breast, lung, and colon cancer) with further subcategorization based on content such as treatment, diagnosis, or other. ChatGPT was deemed to have responded correctly if it chose the correct answer as defined and provided by ASCO-SEP. The study team did not define or determine the correct answer. The program was not asked to provide justifications or references for answers. No point was assigned if ChatGPT-3.5 provided an answer that was not from the options given. Questions where ChatGPT-3.5 chose 2 possible answers or chose multiple answers and did not commit to a single best answer were also considered wrong, even if 1 of the responses was correct.

For statistical analysis, data were logged, scored, and analyzed in Excel (Microsoft Corp). Specifically, a chi-square test was performed to determine if there was a significant difference in the distribution of correct answers across different categories or groups.

## Results

A total of 1040 questions were extracted from the ASCO-SEP question bank.

The questions covered 15 cancer types or disciplines. The largest portion focused on breast (223/1040; 21.4%) and gastrointestinal (209/1040; 20%) cancers, with ≤1% (13/1040) covering central nervous system malignancies, developmental therapeutics, and prevention/epidemiology (Table 1).

**Table 1:** Question distribution and proportions by cancer type or specialty area.

Cancer type or discipline	Number of questions (N=1040), n (%)
Breast cancer	223 (21.4)
Gastrointestinal cancer	209 (20)
Thoracic oncology	137 (13.1)
Hematological malignancies	121 (11.6)
Genitourinary cancer	97 (9)
Melanoma and skin cancer	43 (4)
Sarcoma	36 (3)
Head and neck	36 (3)
Gynecologic cancers	36 (3)
General oncology	29 (3)
Supportive and palliative care	28 (3)
Genetics and genomics	17 (2)
Central nervous system	13 (1)
Developmental therapeutics	10 (1)
Prevention and epidemiology	5 (0.5)

Varying levels of accuracy were observed in ChatGPT-3.5’s performance in answering questions based on different cancer types or disciplines (Table 2). The highest accuracy was achieved in questions related to developmental therapeutics (8/10; 80% correct), while the lowest accuracy was observed for questions related to gastrointestinal cancer (102/209; 48.8% correct).

**Table 2:** Accuracy rates by cancer type or specialty area.

Cancer type or discipline	Discipline-specific accuracy rates, n/N (%)
Developmental therapeutics	8/10 (80)
Central nervous system	10/13 (77)
Melanoma and skin cancer	28/43 (65)
Genetics and genomics	11/17 (65)
General oncology	18/29 (62)
Gynecologic cancers	22/36 (61)
Supportive and palliative care	17/28 (61)
Prevention and epidemiology	3/5 (60)
Head and neck	21/36 (58)
Breast cancer	130/223 (58.3)
Sarcoma	20/36 (57)
Thoracic oncology	77/137 (56)
Hematological malignancies	66/121 (55)
Genitourinary cancer	49/97 (51)
Gastrointestinal cancer	102/209 (48.8)
Total	583/1040 (56.1)

Questions were further subcategorized as “diagnosis,” “treatment,” and “other,” with the latter covering topics such as biostatistics, cancer staging, and treatment complications. Out of the total questions, 73.1% (760/1040) were related to cancer treatment, 10% (99/1040) focused on diagnosis, and the remaining 17.4% (181/1040) were categorized as “other” (Table 3). Accuracy based on subcategory also varied, with 55% (418/760) of treatment questions, 63% (62/99) of diagnosis questions, and 56.9% (103/181) of “other” questions answered correctly (Table 2). There was no significant difference in the program’s performance across the predefined subcategories of diagnosis, treatment, and other (*P*=.16, which is greater than .05).

**Table 3:** ChatGPT-3.5 performance on questions per subcategory.

Category	Number of questions, n (%)	Overall accuracy, n/N (%)	*P* value^a^
Treatment	760 (73.1)	418/760 (55)	.16
Diagnosis	99 (10)	62/99 (63)	.16
Other	181 (17.4)	103/181 (56.9)	.16
Overall	1040 (100)	583/1040 (56.3)	.16

^a^Chi-square test.

Overall, ChatGPT-3.5 achieved a score of 56.3% (583/1040) for correct answers provided across all categories. Of note, responses were marked as incorrect if ChatGPT-3.5 provided 2 or more answers, even if 1 of those answers was correct (37/1040, 3%; Figure 1).

## Discussion

### Overview

In this study, we evaluated the performance of ChatGPT-3.5 in answering ASCO-SEP questions designed for medical oncologists in training and practice to support licensure and ongoing medical education. To facilitate a fair and rigorous assessment, spot checks were performed to ensure answers were not present in the program training data, and questions were entered in separate sessions to avoid grounding bias. Furthermore, questions were presented in their original format, as seen by physicians, with no changes made to prompt the program.

Over 1000 questions were posed to the program, spanning the spectrum of cancer care, with an overall score of 56.3% (583/1040) achieved. While promising, this is, however, below the accepted threshold of 70% that is required by ASCO-SEP to claim CME credits using their question bank [18].

Since the launch of ChatGPT-3.5, several studies have evaluated the program’s performance on medical examinations. A notable study conducted by Kung et al [3] assessed ChatGPT-3.5’s performance on the USMLE taken by US medical students. The results showed that ChatGPT-3.5 performed at, or near, the passing threshold for all 3 examinations. Specifically, the accuracy rates for USMLE Steps 1, 2 CK, and 3 were 68.0%, 58.3%, and 62.4%, respectively, which are acceptable passing scores. Gilson et al [19] reported similar results, where ChatGPT-3.5 scored 60% on USMLE test questions. It is worth noting that although the authors used questions published on the USMLE website after the training date cutoff for ChatGPT, which is late 2021, many of these questions were similar to those published in previous years. Moreover, these questions were discussed on web-based forums, which may explain the higher scores achieved [20]. Additionally, previous studies have evaluated ChatGPT-3.5’s performance in microbiology [21] and pathology [22] and have shown promising outcomes in these fields with an accuracy rate of 80%.

Several factors might explain why ChatGPT’s performs differently on USMLE compared to ASCO-SEP questions. First, the ASCO-SEP is tailored for medical oncologists, delving deep into cancer care, while USMLE caters to a broader set of medical students, covering general medical knowledge. Given that ChatGPT-3.5’s training data spans a wide range of topics, it’s plausible that the content aligns more with the generalized medical queries of USMLE than the specialized focus of ASCO-SEP. Additionally, the structure and phrasing of questions play a critical role, potentially influencing AI’s response accuracy. The questions within the USMLE typically features keywords that assist students in selecting an answer from the provided options. Conversely, the ASCO-SEP presents more specialized questions, challenging physicians’ ability to discern first- and second-line treatments for a specified condition [23]. For instance, in 1 of the numerous subreddits [24] available web-based that was likely included in ChatGPT’s training data set [25] students discuss how certain keywords aid them in answering examination questions. These data might have assisted ChatGPT in responding to USMLE questions in a previous paper that tested ChatGPT’s performance on the USMLE [3,19]. However, such keywords are not used or discussed among physicians engaging with ASCO-SEP questions.

There are additional possible explanations for the observed performance of ChatGPT-3.5 in this study. One key factor is the comprehensive data set of over 1000 questions used, which allowed for a more thorough and holistic evaluation of the program’s performance compared to previous studies [3,19,26,27]. Another contributing factor may be the dynamic and rapid scientific and clinical advances that occur in the field of oncology, which ChatGPT-3.5 could not fully tackle given that its training data is limited to pre-2022 internet data, with restricted access to key databases in the field like PubMed [28].

ChatGPT-3.5 demonstrated varying levels of accuracy in answering questions across the different cancer types and disciplines. Questions related to developmental therapeutics had the highest accuracy rate (80%, 8/10); however, the limited question sample size may not have allowed a complete assessment. Indeed, ChatGPT-3.5’s lowest score was achieved in gastrointestinal cancer, which contained one of the largest numbers of questions in the bank (102/209, 48.8%), suggesting that broader assessments may identify more knowledge gaps. This study did not, however, find any significant difference in ChatGPT-3.5’s performance across the subcategories of diagnosis, treatment, and others.

While ChatGPT-3.5 is not yet fully dependable for complex decision-making in medical oncology, it shows promise in the field. In recent years, we have witnessed significant progress in neural networks, and the future of health care is becoming increasingly multimodal. Oncologists now rely on more than just text-based information when prescribing treatments. They consider a wide range of factors, including diverse image types, genomic data, and social determinants of health. However, in the past, developing multimodal machine learning models seemed like an overly ambitious goal. Thankfully, the landscape has changed, and we have seen exciting advancements in this area through various publications in 2022 and 2023 [29,30]. These studies have showcased the potential applications of multimodal models in the field of oncology, bringing us closer to a more comprehensive and holistic approach to cancer care.

Based on its performance in this study, we do not think that AI can aid oncologists in clinical decision-making at this time. However, it may excel in other tasks in the field [31]. Experts might look to language-generating AI to reduce the burden on humans who create questions and explanations for tests. However, it should be noted that ChatGPT-3.5 is not a useful tool without human supervision at this point, given its potential to fabricate references that may sound plausible but are incorrect [14,32,33]. Oncologists can also use it for administrative tasks such as drafting notes [34] or crafting communication messages for patients [11]. Additionally, while a previous study by Johnson et al [35] demonstrated that ChatGPT can be used by patients to answer common cancer myths and questions, the questions used in this study were already featured on the National Cancer Institute’s webpage and were likely part of ChatGPT’s training data [25] and fewer questions were used. We can infer from this study that the answers provided by ChatGPT still require review by an oncologist to ascertain their accuracy.

In the future, AI has the potential to assist oncologists in critical aspects such as determining optimal chemotherapy dosages [36] and aiding in diagnostics within fields like radiology and pathology [37]. By leveraging the capabilities of these advanced language models, health care professionals can access valuable insights and support in making informed decisions regarding treatment plans. Moreover, patients can also reap the advantages of AI-driven technologies by receiving more accurate diagnoses and tailored treatment approaches, ultimately leading to improved outcomes and enhanced patient care [38].

This study does, however, have several important limitations. First, as ASCO-SEP only consists of MCQs, we did not challenge ChatGPT-3.5 with any other question formats (eg, open-ended), which may have yielded different results. Furthermore, MCQs may not fully reflect the complexity of clinical scenarios that oncologists face in their practice. Second, we did not test the variability of the answers provided by ChatGPT. Each question was presented to ChatGPT 3.5 only once, and the first answer was scored given that previous studies showed high consistency of ChatGPT answers [39] Finally, we could have performed a qualitative assessment of ChatGPT-3.5 answers to gain insights into the etiology of its errors as a guide to future required improvements.

### Conclusions

In conclusion, this study explored the capacity of ChatGPT-3.5’s knowledge in medical oncology using the ASCO-SEP. We aimed to bridge the knowledge gaps surrounding the efficacy of AI-driven tools like ChatGPT-3.5 in supporting clinical decision-making. Our assessment revealed that while ChatGPT-3.5 shows promise for the future of AI in oncology, its current performance on ASCO-SEP underscores a pressing need for further refinement to meet the competency standards in this complex field.

Future evaluations of ChatGPT could extend to assessing its capability in clinical decision support, gauging its accuracy in real-life clinical scenarios, and its ease of integration into medical workflows. Evaluating GPT-4 as a resource to aid oncologists in clinical decision-making, an aspect not available during the tenure of this study, could significantly contribute to the field. The tool’s facilitation of interdisciplinary collaboration among health care professionals and its impact on patient engagement and communication are other potential areas of investigation.

## Abbreviations

AI: artificial intelligence
ASCO-SEP: American Society of Clinical Oncology Self-Evaluation Program
MCQ: multiple choice question
USMLE: United States Medical Licensing Examination

## References

[1] Davenport T, Kalakota R (2019). The potential for artificial intelligence in healthcare. Future Healthc J.

[2] Brown T, Mann B, Ryder N, Subbiah M, Kaplan JD, Dhariwal P, Neelakantan A, Shyam S, Sastry G, Askell A, Agarwal S, Herbert-Voss A, Krueger G, Henighan T, Child R, Ramesh A, Ziegler D, Wu J, Winter C, Hesse C, Chen M, Sigler E, Litwin M, Gray S, Chess B, Clark J, Berner C, McCandlish S, Radford A, Sutskever I, Amodei D (2020). Language models are few-shot learners. Adv Neural Inf Process Syst.

[3] Kung TH, Cheatham M, Medenilla A, Sillos C, De Leon L, Elepaño C, Madriaga M, Aggabao R, Diaz-Candido G, Maningo J, Tseng V (2023). Performance of ChatGPT on USMLE: potential for AI-assisted medical education using large language models. PLOS Digit Health.

[4] (2023). CHatGPT goes law school. University of Minnesota Law School.

[5] Khan RA, Jawaid M, Khan AR, Sajjad M (2023). ChatGPT—reshaping medical education and clinical management. Pak J Med Sci.

[6] King MR (2023). A conversation on artificial intelligence, chatbots, and plagiarism in higher education. Cell Mol Bioeng.

[7] (2023). Large language models. Wikipedia.

[8] Bajwa J, Munir U, Nori A, Williams B (2021). Artificial intelligence in healthcare: transforming the practice of medicine. Future Healthc J.

[9] Alfertshofer M, Hoch CC, Funk PF, Hollmann K, Wollenberg B, Knoedler S, Knoedler L (2023). Sailing the seven seas: a multinational comparison of ChatGPT's performance on medical licensing examinations. Ann Biomed Eng.

[10] Asch D (2023). An interview with ChatGPT about health care. NEJM Catal Innov Care Deliv.

[11] Dave T, Athaluri SA, Singh S (2023). ChatGPT in medicine: an overview of its applications, advantages, limitations, future prospects, and ethical considerations. Front Artif Intell.

[12] Rich AS, Gureckis TM (2019). Lessons for artificial intelligence from the study of natural stupidity. Nat Mach Intell.

[13] Frey CB, Osborne MA (2017). The future of employment: how susceptible are jobs to computerisation?. Technol Forecast Soc Change.

[14] Homolak J (2023). Opportunities and risks of ChatGPT in medicine, science, and academic publishing: a modern promethean dilemma. Croat Med J.

[15] Melnikov AA, Nautrup HP, Krenn M, Dunjko V, Tiersch M, Zeilinger A, Briegel HJ (2018). Active learning machine learns to create new quantum experiments. Proc Natl Acad Sci U S A.

[16] van Dis EAM, Bollen J, Zuidema W, van Rooij R, Bockting CL (2023). ChatGPT: five priorities for research. Nature.

[17] (2023). ASCO-SEP®. American Society of Clinical Oncology® Store.

[18] (2023). ASCO-SEP 6th edition self-evaluation. American Society of Clinical Oncology® Education.

[19] Gilson A, Safranek CW, Huang T, Socrates V, Chi L, Taylor RA, Chartash D (2023). How does ChatGPT perform on the United States medical licensing examination? The implications of large language models for medical education and knowledge assessment. JMIR Med Educ.

[20] (2023). Explanations for the 2020-2022 official step 2 CK practice questions. Ben White.

[21] Das D, Kumar N, Longjam LA, Sinha R, Roy AD, Mondal H, Gupta P (2023). Assessing the capability of ChatGPT in answering first- and second-order knowledge questions on microbiology as per competency-based medical education curriculum. Cureus.

[22] Sinha RK, Roy AD, Kumar N, Mondal H (2023). Applicability of ChatGPT in assisting to solve higher order problems in pathology. Cureus.

[23] Chan MW, Eppich WJ (2020). The keyword effect: a grounded theory study exploring the role of keywords in clinical communication. AEM Educ Train.

[24] (2023). Keywords/Buzzwords on step. Reddit.

[25] Schade M (2023). How ChatGPT and our language models are developed. OpenAI.

[26] Takagi S, Watari T, Erabi A, Sakaguchi K (2023). Performance of GPT-3.5 and GPT-4 on the Japanese medical licensing examination: comparison study. JMIR Med Educ.

[27] Kumah-Crystal Y, Mankowitz S, Embi P, Lehmann CU (2023). ChatGPT and the clinical informatics board examination: the end of unproctored maintenance of certification?. J Am Med Inform Assoc.

[28] Arif TB, Munaf U, Ul-Haque I (2023). The future of medical education and research: Is ChatGPT a blessing or blight in disguise?. Med Educ Online.

[29] Boehm KM, Khosravi P, Vanguri R, Gao J, Shah SP (2022). Harnessing multimodal data integration to advance precision oncology. Nat Rev Cancer.

[30] Foersch S, Glasner C, Woerl AC, Eckstein M, Wagner DC, Schulz S, Kellers F, Fernandez A, Tserea K, Kloth M, Hartmann A, Heintz A, Weichert W, Roth W, Geppert C, Kather JN, Jesinghaus M (2023). Multistain deep learning for prediction of prognosis and therapy response in colorectal cancer. Nat Med.

[31] Liu J, Wang C, Liu S (2023). Utility of ChatGPT in clinical practice. J Med Internet Res.

[32] (2023). David Smerdon. X.

[33] (2023). Teresa Kubacka. X.

[34] Patel SB, Lam K (2023). ChatGPT: the future of discharge summaries?. Lancet Digit Health.

[35] Johnson SB, King AJ, Warner EL, Aneja S, Kann BH, Bylund CL (2023). Using ChatGPT to evaluate cancer myths and misconceptions: artificial intelligence and cancer information. JNCI Cancer Spectr.

[36] Londhe VY, Bhasin B (2019). Artificial intelligence and its potential in oncology. Drug Discov Today.

[37] Luchini C, Pea A, Scarpa A (2022). Artificial intelligence in oncology: current applications and future perspectives. Br J Cancer.

[38] Huang S, Cai N, Pacheco PP, Narrandes S, Wang Y, Xu W (2018). Applications of Support Vector Machine (SVM) learning in cancer genomics. Cancer Genomics Proteomics.

[39] Suárez A, García VDF, Algar J, Gómez Sánchez M, de Pedro ML, Freire Y (2023). Unveiling the ChatGPT phenomenon: evaluating the consistency and accuracy of endodontic question answers. Int Endod J.

